# Systemic Inflammation Response Index (SIRI) and Aggregate Index of Systemic Inflammation (AISI) as Predictors of Mortality in Patients with Upper Gastrointestinal Bleeding

**DOI:** 10.3390/jcm15062245

**Published:** 2026-03-16

**Authors:** Çağdaş Erdoğan, Bayram İnan, İhsan Ateş, Zeki Mesut Yalın Kılıç

**Affiliations:** 1 Department of Gastroenterology, Istinye University, 34485 Istanbul, Turkey; 2 Department of Gastroenterology, Ankara Bilkent Şehir Hastanesi, 06800 Ankara, Turkey; bayraminan84@gmail.com (B.İ.);; 3 Department of Internal Medicine, Ankara Bilkent Şehir Hastanesi, 06800 Ankara, Turkey; dr.ihsanates@hotmail.com

**Keywords:** upper gastrointestinal hemorrhage, systemic inflammation response index, aggregate index of systemic inflammation, prognosis, risk assessment

## Abstract

**Background/Objectives**: Systemic inflammatory markers have recently gained attention as prognostic indicators in various acute conditions. However, their predictive value in non-variceal upper gastrointestinal bleeding (UGIB) remains uncertain. This study aimed to evaluate the prognostic performance of the Systemic Inflammation Response Index (SIRI) and the Aggregate Index of Systemic Inflammation (AISI) for in-hospital mortality among patients with non-variceal UGIB and to compare them with established clinical scoring systems. **Methods**: This retrospective cohort study included 531 adult patients admitted with non-variceal UGIB between April 2023 and February 2025. Demographic, clinical, and laboratory data were collected at presentation. Inflammatory indices (SIRI, AISI, AISI/Hb) and established risk scores (Glasgow-Blatchford, Rockall, AIMS-65, and ABC) were calculated. The primary outcome was all-cause in-hospital mortality. Discriminatory ability was assessed using receiver operating characteristic (ROC) curve analysis, and independent predictors were identified by multivariable logistic regression. **Results**: The overall in-hospital mortality rate was 4.7% (25/531). Non-survivors were older and had lower systolic blood pressure, higher serum urea, and elevated inflammatory indices. Among biomarkers, SIRI (AUC = 0.773, 95% CI: 0.737–0.809) and AISI (AUC = 0.709, 95% CI: 0.670–0.747) showed good discriminatory ability, comparable to AIMS-65 (AUC = 0.765) and ABC (AUC = 0.786). In multivariable models, SIRI (OR = 1.10, *p* = 0.011) and AISI (OR = 1.04 per 100 units, *p* = 0.003) remained independent predictors of mortality after adjustment for age, systolic blood pressure, hemoglobin, serum urea, and albumin. **Conclusions**: SIRI and AISI are independent predictors of in-hospital mortality in patients with non-variceal UGIB, demonstrating comparable prognostic performance to conventional risk scores. These readily available inflammatory indices may serve as simple and cost-effective adjuncts for early risk stratification in clinical practice.

## 1. Introduction

Acute non-variceal upper gastrointestinal bleeding remains a major cause of emergency hospital admission worldwide. Despite advances in endoscopic therapy and supportive management, it continues to be associated with considerable morbidity and mortality, particularly among older patients and those with significant comorbidities. Epidemiological studies report an annual incidence ranging from 48 to 160 cases per 100,000 population and in-hospital mortality rates of approximately 2–10% [1,2,3,4].

Early risk assessment at presentation is essential for guiding triage decisions, including level of care, timing of endoscopy, and therapeutic strategy. Several prognostic models have therefore been developed, such as the Glasgow-Blatchford, Rockall, AIMS-65, and ABC scores. Although these tools combine clinical and laboratory parameters to estimate outcomes, their performance varies across clinical settings. The Rockall score requires endoscopic findings and is therefore less applicable before endoscopy, whereas the Glasgow-Blatchford score is more effective for identifying low-risk patients than for predicting mortality [5,6,7].

Growing evidence suggests that the systemic inflammatory response plays an important role in the pathophysiology and clinical course of upper gastrointestinal bleeding. Leukocyte subtypes—particularly neutrophils, monocytes, and lymphocytes—reflect the balance between pro- and anti-inflammatory pathways activated during hemorrhagic stress and tissue injury. Accordingly, composite indices derived from routine blood counts have been investigated as accessible markers of disease severity and prognosis in acute conditions [8,9].

Among composite inflammatory biomarkers, the systemic inflammation response index (SIRI)—calculated from neutrophil, monocyte, and lymphocyte counts—integrates key components of both innate and adaptive immunity. Elevated SIRI levels have been associated with adverse outcomes in diverse clinical contexts, including cerebrovascular disease, cardiovascular risk populations, and malignancies, supporting its value as a global indicator of inflammatory burden [10,11,12].

The aggregate index of systemic inflammation (AISI) extends this concept by additionally incorporating platelet counts, thereby capturing interactions between inflammatory and thrombotic pathways. Increased AISI values have been linked to mortality and disease progression in several chronic and acute disorders. By integrating multiple hematologic lineages simultaneously, AISI may provide a more comprehensive representation of the host response to physiological stress [13,14,15].

Despite the established prognostic value of SIRI and AISI in various clinical contexts, their utility in predicting mortality among patients with non-variceal UGIB has not been systematically evaluated. Given the critical role of systemic inflammation in determining outcomes and the readily available nature of these indices, they may represent valuable additions to existing risk stratification tools. The present study aims to investigate the prognostic significance of SIRI and AISI in predicting in-hospital mortality among patients presenting with non-variceal UGIB and to compare their discriminatory ability with established clinical scoring systems. In addition, we explored AISIHb, a modified composite index incorporating hemoglobin, to examine whether integrating anemia—a key determinant of bleeding severity and tissue hypoxia—into inflammatory indices may provide additional pathophysiological insight in UGIB. To date, the prognostic performance of these composite inflammatory indices has not been systematically evaluated in patients with non-variceal UGIB or directly compared with established clinical risk stratification systems in a large clinical cohort.

## 2. Materials and Methods

### 2.1. Study Design and Setting

We performed a retrospective observational cohort study at Ankara Bilkent City Hospital, a tertiary academic referral center in Ankara, Turkey. Adult patients presenting with suspected non-variceal upper gastrointestinal bleeding between April 2023 and February 2025 were screened for eligibility. The study protocol was approved by the institutional ethics committee (TABED 26 February 1947) and conducted in accordance with the Declaration of Helsinki. The requirement for informed consent was waived due to the retrospective design.

### 2.2. Study Population

All consecutive adult patients (≥18 years of age) who presented with signs and symptoms of suspected non-variceal upper gastrointestinal bleeding (non-variceal UGIB) were eligible for inclusion. Non-variceal UGIB was defined as hematemesis, melena, or hematochezia with an upper gastrointestinal source confirmed by esophagogastroduodenoscopy (EGD) and absence of esophageal or gastric variceal bleeding. Patients were excluded if they had incomplete medical records or missing laboratory data at presentation. Endoscopic evaluation within 24 h was not mandatory, as clinically suspected non-variceal UGIB was adjudicated based on presenting symptoms and physician documentation in patients who died early or were not stable enough to undergo endoscopy. During the study period, a total of 634 patients with suspected non-variceal UGIB were screened. After exclusion of patients with missing admission laboratory data (*n* = 46), incomplete medical records (*n* = 31), or alternative non-UGIB diagnoses (*n* = 26), 531 patients were included in the final analysis. In patients who did not undergo endoscopy, the diagnosis of non-variceal UGIB was adjudicated retrospectively based on documented hematemesis, melena, and hemodynamic instability consistent with upper gastrointestinal bleeding, and treating physician assessment No imputation was performed for missing variables; cases with missing baseline laboratory parameters required for inflammatory index calculation were excluded.

### 2.3. Data Collection

Demographic and clinical data were extracted from the hospital’s electronic medical records system. The following variables were collected: age, sex, vital signs at presentation (systolic and diastolic blood pressure, heart rate), comorbidities, medication history (including antiplatelet and anticoagulant use), and clinical presentation (hematemesis, melena, syncope). Laboratory parameters obtained at the time of initial presentation included complete blood count (hemoglobin, hematocrit, white blood cell count with differential, and platelet count), serum biochemistry (albumin, blood urea nitrogen [BUN], creatinine), and international normalized ratio (INR). Endoscopic findings were recorded according to the Forrest classification for peptic ulcer bleeding, and other diagnoses were categorized as gastritis, esophagitis, Mallory–Weiss tear, angiodysplasia, or normal endoscopy.

### 2.4. Inflammatory Indices and Risk Scores

Several hematological inflammatory indices were calculated from the initial complete blood count:

- Neutrophil-to-lymphocyte ratio (NLR) = neutrophil count/lymphocyte count;
- Platelet-to-lymphocyte ratio (PLR) = platelet count/lymphocyte count;
- Monocyte-to-lymphocyte ratio (MLR) = monocyte count/lymphocyte count;
- Systemic immune-inflammation index (SII) = (neutrophil count × platelet count)/lymphocyte count;
- Systemic inflammation response index (SIRI) = (neutrophil count × monocyte count)/lymphocyte count;
- Aggregate index of systemic inflammation (AISI) = (neutrophil count × monocyte count × platelet count)/lymphocyte count.

Additionally, the AISI-to-hemoglobin ratio (AISI/Hb) was calculated as a novel combined parameter.

Established clinical risk stratification scores were calculated for all patients, including the Glasgow-Blatchford Score (GBS), pre-endoscopy Rockall Score, AIMS65 Score, and ABC Score. These scores were computed based on their respective validated formulas using clinical and laboratory parameters at presentation.

### 2.5. Outcome Measures

The primary outcome was all-cause in-hospital mortality. Secondary outcomes included the need for intensive care unit (ICU) admission, blood transfusion requirements (number of packed red blood cell units), endoscopic hemostasis intervention, rebleeding during hospitalization, and length of hospital stay. Mortality was defined as death occurring during the index hospitalization for non-variceal UGIB, regardless of the cause.

### 2.6. Statistical Analysis

All statistical analyses were conducted using Python (version 3.12.0) with the pandas, NumPy, SciPy, scikit-learn, statsmodels, and lifelines libraries. Continuous variables were expressed as mean ± standard deviation or median with interquartile range, as appropriate. Categorical variables were presented as frequencies and percentages. The Shapiro–Wilk test was used to assess normality of distribution.

For comparison of baseline characteristics between survivors and non-survivors, the Mann–Whitney U test was used for continuous variables, and Fisher’s exact test or chi-square test was applied for categorical variables. Inflammation scores were compared between groups using the Mann–Whitney U test, and fold changes were calculated as the ratio of median values (non-survivors/survivors).

Receiver operating characteristic (ROC) curve analysis was performed to evaluate the discriminatory ability of inflammation scores and clinical scoring systems for predicting mortality. The area under the curve (AUC) with 95% confidence intervals (CI) was calculated. Optimal cutoff values were determined using the Youden index, and sensitivity, specificity, positive predictive value (PPV), negative predictive value (NPV), and accuracy were computed.

Multivariable logistic regression analysis was conducted to identify independent predictors of mortality. Given the limited number of mortality events, the number of covariates included in multivariable models was restricted based on established clinical relevance and prior UGIB prognostic literature, in addition to univariable screening (*p* < 0.10), to reduce the risk of overfitting. Odds ratios (OR) with 95% CI were reported. Model performance was assessed using McFadden’s pseudo R^2^ and likelihood ratio test.

Pearson correlation coefficients were calculated to assess the relationships between inflammation scores and clinical scoring systems. Kaplan–Meier survival analysis was performed to compare survival probabilities between groups stratified by optimal cutoff values, and the log-rank test was used to evaluate differences between survival curves.

All statistical tests were two-sided, and a *p*-value < 0.05 was considered statistically significant. Data visualization was performed using Matplotlib (3.10.7) and Seaborn 0.13.2 libraries.

## 3. Results

### 3.1. Baseline Characteristics and Endoscopic Findings

Among 634 patients screened during the study period, 531 met the inclusion criteria and were included in the final analysis. A total of 531 patients with upper gastrointestinal bleeding were included in the study. The overall in-hospital mortality rate was 4.7% (25/531). Baseline characteristics of patients stratified by mortality status are presented in Table 1. Among the 59 patients who did not undergo endoscopy, most died early after admission or were hemodynamically unstable; therefore, UGIB diagnosis in these cases was based on clinical adjudication rather than endoscopic confirmation.

Non-survivors were significantly older than survivors (74.0 ± 18.4 vs. 63.7 ± 19.5 years, *p* = 0.006). Regarding clinical presentation, hematochezia (36.0% vs. 10.5%, *p* = 0.001) and syncope (28.0% vs. 9.7%, *p* = 0.011) were more frequent in non-survivors. Vital signs at admission showed that non-survivors had lower systolic blood pressure (106.4 ± 25.6 vs. 124.0 ± 19.2 mmHg, *p* < 0.001) and diastolic blood pressure (60.7 ± 16.0 vs. 69.5 ± 11.6 mmHg, *p* = 0.011).

Laboratory parameters revealed that non-survivors had significantly higher white blood cell counts (13.48 ± 7.57 vs. 9.46 ± 3.80 ×10^3^/μL, *p* = 0.002), neutrophil counts (11.30 ± 6.95 vs. 7.03 ± 3.51 ×10^3^/μL, *p* < 0.001), blood urea nitrogen (9.23 ± 5.19 vs. 5.67 ± 3.86 mg/dL, *p* < 0.001), creatinine (1.49 ± 0.75 vs. 1.02 ± 0.42 mg/dL, *p* = 0.001), and C-reactive protein (85.66 ± 108.59 vs. 17.61 ± 35.00 mg/L, *p* < 0.001). Conversely, non-survivors had lower lymphocyte counts (1.24 ± 0.66 vs. 1.68 ± 0.89 ×10^3^/μL, *p* = 0.010) and albumin levels (3.12 ± 0.46 vs. 3.81 ± 0.53 g/dL, *p* < 0.001). Clinical scoring systems including Glasgow-Blatchford Score (8.60 ± 4.27 vs. 5.96 ± 4.15, *p* = 0.003), AIMS-65 Score (1.84 ± 1.11 vs. 0.80 ± 0.80, *p* < 0.001), and ABC Score (5.24 ± 2.57 vs. 2.59 ± 2.06, *p* < 0.001) were significantly higher in non-survivors.

Endoscopy was performed in 88.9% (472/531) of all patients. Notably, the proportion of patients who underwent endoscopy was significantly lower in non-survivors compared to survivors (52.0% vs. 90.7%, *p* < 0.001). Among patients who underwent endoscopy, the most common finding was gastritis (38.1%), followed by Forrest 3 ulcer with clean base (25.4%). The distribution of endoscopic findings did not differ significantly between survivors and non-survivors. Most non-survivors who did not undergo endoscopy died early after admission or were clinically unstable and therefore not considered suitable for endoscopic evaluation.

### 3.2. Comparison of Inflammation Scores

All inflammation scores except platelet-to-lymphocyte ratio (PLR) were significantly elevated in non-survivors compared to survivors (Table 2). The systemic inflammation response index (SIRI) demonstrated a 2.38-fold increase in non-survivors (median 5.08 vs. 1.79, *p* < 0.001). The aggregate index of systemic inflammation (AISI) showed a 2.28-fold increase (median 1129.08 vs. 473.56, *p* < 0.001), while AISI adjusted for hemoglobin (AISI/Hb) exhibited the highest fold change at 2.93 (median 92.55 vs. 46.48, *p* < 0.001). The neutrophil-to-lymphocyte ratio (NLR) increased 2.30-fold (median 9.23 vs. 4.02, *p* < 0.001), and the systemic immune-inflammation index (SII) showed a 1.68-fold increase (median 1793.35 vs. 1067.14, *p* < 0.001).

### 3.3. Predictive Performance for Mortality

**Figure 1:**
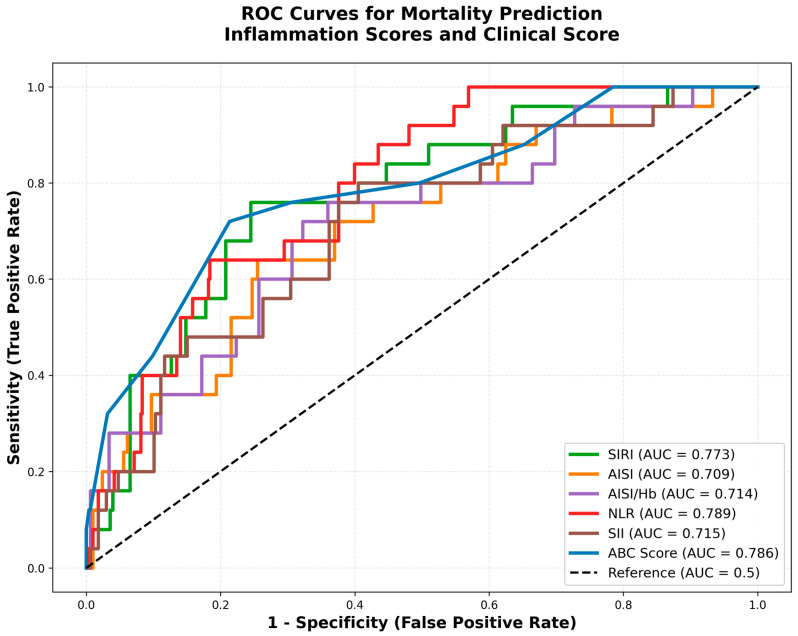
Receiver operating characteristic (ROC) curves for mortality prediction. ROC curves showing the discriminatory ability of inflammation-based scores and clinical scoring systems for predicting in-hospital mortality. The curves represent SIRI (AUC = 0.773), AISI (AUC = 0.709), AISI/Hb (AUC = 0.714), NLR (AUC = 0.789), SII (AUC = 0.715), and ABC Score (AUC = 0.786). The diagonal reference line represents AUC = 0.5 (no discrimination). SIRI, systemic inflammation response index; AISI, aggregate index of systemic inflammation; Hb, hemoglobin; NLR, neutrophil-to-lymphocyte ratio; SII, systemic immune-inflammation index; ABC, Age–Blood–Comorbidity Score.

ROC curve analysis revealed that NLR had the highest discriminatory ability for predicting mortality (AUC = 0.789, 95% CI: 0.754–0.823), followed by ABC Score (AUC = 0.786, 95% CI: 0.751–0.821) and SIRI (AUC = 0.773, 95% CI: 0.737–0.809) (Table 3, Figure 1). The optimal cutoff value for SIRI was 3.49, yielding a sensitivity of 76.0% and specificity of 75.5%. AISI demonstrated an AUC of 0.709 (95% CI: 0.670–0.747) with an optimal cutoff of 956.31 (sensitivity 64.0%, specificity 74.5%). AISI/Hb showed an AUC of 0.714 (95% CI: 0.675–0.752). Among established clinical scoring systems, AIMS-65 achieved an AUC of 0.765, while Glasgow-Blatchford Score and Rockall Score showed lower discriminatory abilities (AUC = 0.673 and 0.594, respectively).

### 3.4. Independent Predictors of Mortality

Multivariable logistic regression analysis identified SIRI as independently associated with mortality after adjusting for age, sex, initial hemoglobin, systolic blood pressure, blood urea nitrogen, and albumin (OR = 1.102, 95% CI: 1.022–1.188, *p* = 0.011) (Table 4A). In this model, albumin (OR = 0.060, 95% CI: 0.018–0.203, *p* < 0.001) and systolic blood pressure (OR = 0.971, 95% CI: 0.949–0.993, *p* = 0.011) were also independent predictors. The model achieved a McFadden’s R^2^ of 0.316.

Similarly, AISI remained an independent predictor in a separate model (OR = 1.036 per 100-unit increase, 95% CI: 1.012–1.061, *p* = 0.003) (Table 4B). This model, which also included albumin (OR = 0.054, 95% CI: 0.016–0.184, *p* < 0.001), systolic blood pressure (OR = 0.970, 95% CI: 0.949–0.992, *p* = 0.009), blood urea nitrogen (OR = 1.094, 95% CI: 1.005–1.191, *p* = 0.038), and initial hemoglobin (OR = 1.212, 95% CI: 1.005–1.461, *p* = 0.044), demonstrated a McFadden’s R^2^ of 0.321.

### 3.5. Correlations Between Inflammation Scores

**Figure 2:**
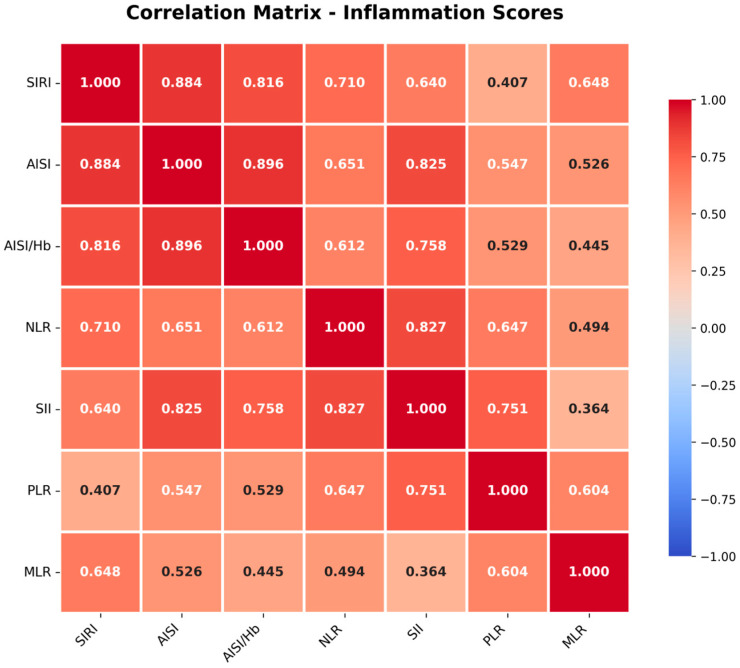
Correlation matrix of inflammation scores. Heat map depicting Pearson correlation coefficients among seven inflammation-based scores. Strong positive correlations (|r| > 0.7, shown in bold) were observed between SIRI and AISI (r = 0.884), AISI and AISI/Hb (r = 0.896), and SIRI and AISI/Hb (r = 0.816). Color intensity indicates correlation strength: red represents positive correlation, blue represents negative correlation. SIRI, systemic inflammation response index; AISI, aggregate index of systemic inflammation; Hb, hemoglobin; NLR, neutrophil-to-lymphocyte ratio; SII, systemic immune-inflammation index; PLR, platelet-to-lymphocyte ratio; MLR, monocyte-to-lymphocyte ratio.

Correlation analysis revealed strong positive correlations among inflammation scores (Figure 2). SIRI showed strong correlations with AISI (r = 0.884), AISI/Hb (r = 0.816), and NLR (r = 0.710). AISI demonstrated strong correlations with AISI/Hb (r = 0.896) and SII (r = 0.825). The correlations between inflammation scores and established clinical scoring systems (Glasgow-Blatchford Score, AIMS-65, ABC Score, and Rockall Score) were weak to moderate, with correlation coefficients ranging from −0.003 to 0.288.

### 3.6. Kaplan–Meier Survival Analysis

**Figure 3:**
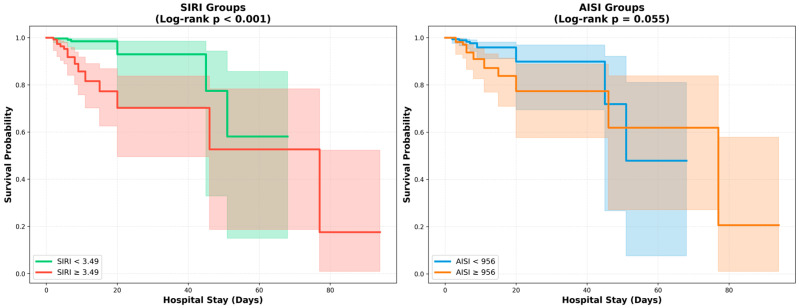
Kaplan–Meier survival curves stratified by inflammation scores. Survival probability curves comparing patients stratified by optimal cutoff values for SIRI (**Left panel**) and AISI (**Right panel**). (**Left**) Patients with SIRI ≥ 3.49 had significantly lower survival compared to those with SIRI < 3.49 (log-rank *p* < 0.001; mortality 13.3% vs. 1.6%). (**Right**) Patients with AISI ≥ 956 showed a trend toward lower survival compared to those with AISI < 956 (log-rank *p* = 0.055; mortality 10.4% vs. 2.6%). Shaded areas represent 95% confidence intervals. SIRI, systemic inflammation response index; AISI, aggregate index of systemic inflammation.

Kaplan–Meier survival analysis stratified by optimal cutoff values demonstrated significant differences in survival between groups (Table 5, Figure 3). Patients with SIRI ≥ 3.49 had significantly lower survival compared to those with SIRI < 3.49 (log-rank *p* < 0.001). The mortality rate was 13.3% in the high SIRI group versus 1.6% in the low SIRI group. For AISI, patients with values ≥ 956.31 showed a trend toward lower survival compared to those with AISI < 956.31 (log-rank *p* = 0.055). The mortality rate was 10.4% in the high AISI group versus 2.6% in the low AISI group.

## 4. Discussion

In this single-center retrospective cohort of 531 patients with acute non-variceal upper gastrointestinal bleeding (non-variceal UGIB), we demonstrated that the Systemic Inflammation Response Index (SIRI) and the Aggregate Index of Systemic Inflammation (AISI) were significantly associated with in-hospital mortality. Notably, SIRI exhibited robust discriminatory ability in ROC analysis and remained independently associated with mortality in multivariable models. Although AISI was also associated with mortality, its prognostic performance was more modest. Patients with elevated SIRI values had significantly higher mortality in Kaplan–Meier analyses, underscoring the potential of SIRI as a clinically useful biomarker for early risk stratification in non-variceal UGIB. Compared with previous studies evaluating isolated inflammatory markers in non-variceal UGIB, our study examines composite indices in a larger clinical cohort and directly benchmarks their prognostic performance against established clinical risk scores. These findings support the potential role of CBC-derived composite inflammatory indices as practical adjuncts to conventional risk stratification tools rather than replacements.

Early risk assessment in non-variceal UGIB is crucial to guide clinical decision-making, including triage, timing of endoscopy, and allocation of intensive care resources. Current clinical practice incorporates validated risk scoring systems such as the Glasgow Blatchford Score (GBS), AIMS65, and Rockall Score, which have been shown to predict rebleeding and mortality across multiple cohorts [3,7]. In a multicenter study, Stanley et al. demonstrated that AIMS65 performed strongly in predicting in-hospital mortality [7]. More recently, Laursen et al. developed and externally validated the ABC score, which showed superior prognostic accuracy compared to conventional models such as AIMS65 and GBS [16]. Despite these advances, there remains a need for novel, readily available parameters to enhance early prognostic assessment, particularly before endoscopic evaluation.

Inflammation plays a central role in the pathophysiology of acute bleeding, influencing hemodynamic stress, tissue injury, and immune response. Composite hematologic ratios derived from complete blood counts have recently emerged as potential markers of disease severity. Chen et al. demonstrated that a higher neutrophil-to-lymphocyte ratio (NLR) was independently associated with adverse outcomes in non-variceal UGIB, supporting the link between systemic inflammation and clinical prognosis [9]. Similarly, Park and colleagues (2020) reported that serum C-reactive protein (CRP) at admission independently predicted mortality in non-variceal UGIB, suggesting that acute-phase reactants reflect the inflammatory burden of critical bleeding [17]. Dertli et al. (2022) also showed in a cohort of 250 patients that higher NLR values were independently associated with in-hospital mortality, emphasizing the reproducibility of inflammation-driven risk estimation [18]. Furthermore, Lee et al. (2015) observed that CRP predicted 30-day rebleeding risk in non-variceal UGIB, linking systemic inflammation to early recurrence of bleeding [19].

Evidence from prospective series supports the complexity of prognostic determinants. Jiménez-Rosales et al. (2018) demonstrated that the determinants of in-hospital versus delayed (6-month) mortality differ substantially, with comorbidity and hypoalbuminemia exerting stronger long-term effects [20]. This aligns with our findings that SIRI and AISI, both incorporating immune and nutritional components, may integrate multiple pathophysiological domains into a single prognostic construct.

Recent investigations have explored inflammation-based indices more directly. Altınsoy and colleagues (2025) examined 182 emergency-department patients with acute gastrointestinal bleeding and showed that elevated systemic immune-inflammation index (SII) levels were independently associated with mortality, outperforming individual markers such as CRP (AUC 0.81) [21]. In contrast, Bozan and Atiş (2022) found no correlation between SII or CRP/albumin ratio and Forrest class among endoscopically confirmed non-variceal UGIB cases, highlighting the distinction between anatomical severity and systemic response [22]. Similarly, Cazacu et al. (2025) reported that NLR predicted survival and rebleeding risk in portal hypertensive non-variceal UGIB, reinforcing that leukocyte-derived ratios capture illness severity across both variceal and non-variceal bleeding [23].

Our findings also align with broader evidence linking SIRI and AISI to poor outcomes in diverse clinical settings. Shi et al. (2024) showed that elevated SIRI predicted all-cause and cardiovascular mortality among hemodialysis patients [24], while Zinellu et al. (2021) demonstrated that AISI was an independent prognostic biomarker in idiopathic pulmonary fibrosis [13]. Moreover, in a recent meta-analysis of seven studies, high SIRI was consistently associated with shorter overall and progression-free survival among pancreatic cancer patients [25]. Together, these studies suggest that composite inflammatory indices—by capturing innate, adaptive, and platelet-driven inflammatory activity—may provide a stable, biologically relevant measure of systemic stress and immune dysregulation. It should be noted that much of the existing evidence on SIRI and AISI derives from non-bleeding conditions such as malignancy, cardiovascular disease, or chronic inflammatory disorders; therefore, direct extrapolation to non-variceal UGIB populations should be interpreted cautiously.

Mechanistically, SIRI (neutrophil × monocyte/lymphocyte) and AISI (neutrophil × monocyte × platelet/lymphocyte) reflect complex interactions among innate and adaptive immune pathways. Elevated neutrophils and monocytes indicate a pro-inflammatory state, while lymphopenia denotes immune suppression, and platelets contribute to thrombo-inflammatory activation. In non-variceal UGIB, acute blood loss, tissue ischemia, and stress-related cytokine release may amplify these pathways, leading to adverse outcomes. Thus, higher SIRI and AISI levels could represent both the magnitude and persistence of systemic inflammation during the bleeding episode. Consistent with their shared hematologic components, strong correlations were observed among composite inflammatory indices; however, these relationships primarily reflect mathematical overlap rather than independent clinical prognostic information. AISIHb was explored as a modified index incorporating hemoglobin to account for the contribution of anemia to bleeding severity and systemic hypoxia. However, its interpretability is inherently more complex than leukocyte-based indices, as hemoglobin reflects both acute blood loss and chronic comorbid conditions. In our cohort, AISIHb did not demonstrate clear incremental prognostic value beyond other inflammatory indices.

Although NLR demonstrated the highest AUC among inflammatory markers in our cohort, SIRI showed comparable discriminatory performance and retained an independent association with mortality in multivariable analysis. The rationale for evaluating composite indices such as SIRI and AISI lies in their integration of multiple inflammatory cell lineages, potentially capturing a broader systemic response than single-ratio markers like NLR. However, the practical incremental value of SIRI over simpler indices such as NLR remains uncertain and requires validation in larger cohorts. Incorporating SIRI into early risk stratification may enhance clinical decision-making, particularly in settings where endoscopic or radiologic assessment is not immediately available. These observations complement earlier work by Isfahani et al. (2025), who showed that pre-endoscopic scoring systems such as AIMS65, GBS, and Rockall could effectively predict mortality and readmission in non-variceal UGIB [26]. Future studies may evaluate whether combining inflammation-based markers with clinical scores yields superior risk discrimination. The relatively low positive predictive values observed across all scores primarily reflect the low in-hospital mortality rate in this cohort (4.7%), as PPV is strongly influenced by outcome prevalence. In contrast, the consistently high negative predictive values indicate that these scores are more effective for identifying low-risk patients rather than predicting death with high certainty, which is typical for prognostic models in UGIB populations. Given that NLR is simpler to calculate and interpret, its clinical utility may remain more practical unless future studies demonstrate consistent superiority of composite indices.

Our study has several strengths, including a relatively large sample size, comprehensive laboratory and clinical data, and multivariable modeling that accounted for relevant confounders. However, several limitations should be considered when interpreting our findings. First, the retrospective and single-center design may limit generalizability to other populations and healthcare settings. Second, the number of mortality events was relatively modest in relation to the number of covariates included in the multivariable models, which may increase the risk of overfitting and limit model stability despite clinically guided variable selection. Therefore, the multivariable findings should be interpreted as exploratory and hypothesis-generating rather than definitive. Third, although we assessed discriminative performance using ROC analysis, we did not evaluate calibration metrics or formally test whether the addition of inflammatory indices improves predictive accuracy beyond established clinical scores. Additionally, CBC-derived inflammatory indices are influenced by various acute and chronic conditions such as infection, systemic inflammatory disorders, malignancy, renal dysfunction, and physiological stress, many of which also contribute to all-cause in-hospital mortality. Because the primary outcome was all-cause mortality regardless of cause, residual confounding unrelated to UGIB severity cannot be excluded. Fourth, endoscopic evaluation was substantially less frequent in non-survivors, primarily because many patients died early after admission or were too hemodynamically unstable to undergo endoscopy. This may have led to underrepresentation of endoscopic severity markers in the highest-risk group. However, excluding these early deaths would have underestimated true in-hospital mortality and distorted the clinical spectrum of severe UGIB. Consequently, we were unable to adjust for endoscopic severity in the highest-risk patients, which limits causal interpretation of the observed associations between inflammatory indices and mortality. These factors should be considered when interpreting the observed independent associations of SIRI and AISI with mortality. Future multicenter studies with larger event numbers and external validation are warranted. Furthermore, similar discriminative ability (AUC) does not necessarily translate into greater clinical utility. We did not assess calibration, reclassification, or decision-analytic metrics; therefore, comparable AUC values between inflammatory indices and established scores should not be interpreted as equivalent clinical usefulness.

## 5. Conclusions

Our study demonstrates that SIRI and AISI are associated with in-hospital mortality in patients with non-variceal UGIB, with SIRI showing an independent association with mortality. These easily obtainable inflammatory indices may complement existing risk scores and improve early prognostic assessment. Further validation across larger, diverse populations and external cohorts is required before these indices can be integrated into routine clinical risk stratification.

## Figures and Tables

**Table 1:** Baseline Characteristics and Endoscopic Findings of Patients Stratified by Mortality Status.

Variable	All Patients (*n* = 531)	Survivors (*n* = 506)	Non-Survivors (*n* = 25)	*p*-Value
Age (years)	64.2 ± 19.6	63.7 ± 19.5	74.0 ± 18.4	**0.006**
Male sex, *n* (%)	313 (58.9%)	303 (59.9%)	10 (40.0%)	0.06
Comorbidities, *n* (%) Hypertension	257 (48.4%)	245 (48.4%)	12 (48.0%)	1.0
Diabetes mellitus	142 (26.7%)	139 (27.5%)	3 (12.0%)	0.106
Coronary artery disease	166 (31.3%)	157 (31.0%)	9 (36.0%)	0.659
Atrial fibrillation	72 (13.6%)	66 (13.0%)	6 (24.0%)	0.132
Cerebrovascular disease	29 (5.5%)	26 (5.1%)	3 (12.0%)	0.15
Medications, n (%) Antiplatelet therapy	170 (32.0%)	159 (31.4%)	11 (44.0%)	0.194
Anticoagulant therapy	126 (23.7%)	116 (22.9%)	10 (40.0%)	0.057
Oral antidiabetic	106 (20.0%)	103 (20.4%)	3 (12.0%)	0.443
Insulin	21 (4.0%)	21 (4.2%)	0 (0.0%)	0.615
Statin	62 (11.7%)	59 (11.7%)	3 (12.0%)	1.0
Presenting symptoms, n (%) Melena	384 (72.3%)	362 (71.5%)	22 (88.0%)	0.106
Hematochezia	62 (11.7%)	53 (10.5%)	9 (36.0%)	**0.001**
Hematemesis	209 (39.4%)	201 (39.7%)	8 (32.0%)	0.532
Syncope	56 (10.5%)	49 (9.7%)	7 (28.0%)	**0.011**
Heart rate (bpm)	87.9 ± 17.9	87.6 ± 17.4	94.2 ± 25.2	0.085
Systolic BP (mmHg)	123.2 ± 19.8	124.0 ± 19.2	106.4 ± 25.6	**0.0**
Diastolic BP (mmHg)	69.1 ± 11.9	69.5 ± 11.6	60.7 ± 16.0	**0.011**
Laboratory findings Initial hemoglobin (g/dL)	10.53 ± 3.04	10.57 ± 3.03	9.60 ± 3.04	0.158
Lowest hemoglobin (g/dL)	9.25 ± 2.71	9.31 ± 2.72	8.04 ± 2.24	0.06
Hemoglobin drop (g/dL)	1.28 ± 1.18	1.26 ± 1.16	1.55 ± 1.46	0.687
White blood cell (×10^3^/μL)	9.65 ± 4.13	9.46 ± 3.80	13.48 ± 7.57	**0.002**
Neutrophil (×10^3^/μL)	7.23 ± 3.84	7.03 ± 3.51	11.30 ± 6.95	**0.0**
Lymphocyte (×10^3^/μL)	1.66 ± 0.89	1.68 ± 0.89	1.24 ± 0.66	**0.01**
Monocyte (×10^3^/μL)	0.52 ± 0.42	0.51 ± 0.42	0.63 ± 0.44	0.121
Platelet (×10^3^/μL)	273.77 ± 104.31	273.17 ± 97.23	285.92 ± 202.99	0.297
Urea (mg/dL)	75.21 ± 51.24	73.03 ± 49.72	118.88 ± 66.85	**0.0**
Creatinine (mg/dL)	1.04 ± 0.45	1.02 ± 0.42	1.49 ± 0.75	**0.001**
Albumin (g/dL)	3.77 ± 0.55	3.81 ± 0.53	3.12 ± 0.46	**0.0**
CRP (mg/L)	20.81 ± 43.69	17.61 ± 35.00	85.66 ± 108.59	**0.0**
Glasgow-Blatchford Score	6.08 ± 4.19	5.96 ± 4.15	8.60 ± 4.27	**0.003**
AIMS-65 Score	0.85 ± 0.84	0.80 ± 0.80	1.84 ± 1.11	**0.0**
ABC Score	2.72 ± 2.15	2.59 ± 2.06	5.24 ± 2.57	**0.0**
Rockall Score	3.65 ± 1.94	3.63 ± 1.92	4.46 ± 2.40	0.243
Endoscopy performed, *n* (%)	472 (88.9%)	459 (90.7%)	13 (52.0%)	**<0.001**
Endoscopy findings, *n* (%) Normal, *n* (%)	20 (4.2%)	20 (4.4%)	0 (0.0%)	1.0
Forrest 1A (active spurting), *n* (%)	4 (0.8%)	4 (0.9%)	0 (0.0%)	1.0
Forrest 1B (active oozing), *n* (%)	29 (6.1%)	27 (5.9%)	2 (15.4%)	0.187
Forrest 2A (visible vessel), *n* (%)	35 (7.4%)	35 (7.6%)	0 (0.0%)	0.612
Forrest 2B (adherent clot), *n* (%)	22 (4.7%)	22 (4.8%)	0 (0.0%)	1.0
Forrest 2C (flat spot), *n* (%)	29 (6.1%)	29 (6.3%)	0 (0.0%)	1.0
Forrest 3 (clean base), *n* (%)	120 (25.4%)	115 (25.1%)	5 (38.5%)	0.331
Gastritis, *n* (%)	180 (38.1%)	174 (37.9%)	6 (46.2%)	0.572
Esophagitis, *n* (%)	27 (5.7%)	27 (5.9%)	0 (0.0%)	1.0
Mallory–Weiss tear, *n* (%)	2 (0.4%)	2 (0.4%)	0 (0.0%)	1.0
Angiodysplasia, *n* (%)	4 (0.8%)	4 (0.9%)	0 (0.0%)	1.0

Data are presented as mean ± standard deviation for continuous variables and *n* (%) for categorical variables. Endoscopic findings are reported among patients who underwent endoscopy (*n* = 472). BP, blood pressure; BUN, blood urea nitrogen; CRP, C-reactive protein. Bold *p*-values indicate statistical significance (*p* < 0.05).

**Table 2:** Comparison of Inflammation Scores Between Survivors and Non-survivors.

Inflammation Score	All Patients	Survivors	Non-Survivors	*p*-Value	Fold Change
NLR	4.15	4.02	9.23	0.0	1.98
PLR	172.2	172.15	248.39	0.101	1.31
LMR	3.3	3.42	2.12	0.0	0.59
MLR	0.3	0.29	0.47	0.0	1.45
SII	1088.27	1067.14	1793.35	0.0	1.87
SIRI	1.86	1.79	5.08	0.0	2.38
AISI	480.48	473.56	1129.08	0.0	2.28
AISI/Hb	48.57	46.48	92.55	0.0	2.93

Data are presented as median values. NLR, neutrophil-to-lymphocyte ratio; PLR, platelet-to-lymphocyte ratio; LMR, lymphocyte-to-monocyte ratio; MLR, monocyte-to-lymphocyte ratio; SII, systemic immune-inflammation index; SIRI, systemic inflammation response index; AISI, aggregate index of systemic inflammation; Hb, hemoglobin. Fold change represents the ratio of median values (non-survivors/survivors). Bold *p*-values indicate statistical significance (*p* < 0.05).

**Table 3:** Receiver Operating Characteristic (ROC) Curve Analysis for Mortality Prediction.

Score	AUC	95% CI	Optimal Cutoff	Sensitivity (%)	Specificity (%)	PPV (%)	NPV (%)	Accuracy (%)
NLR	0.789	0.754–0.823	7.98	64.0%	81.6%	14.7%	97.9%	80.8%
ABC	0.786	0.751–0.821	5.0	72.0%	78.7%	14.3%	98.3%	78.3%
SIRI	0.773	0.737–0.809	3.49	76.0%	75.5%	13.3%	98.5%	75.5%
AIMS-65	0.765	0.729–0.801	2.0	60.0%	84.2%	15.8%	97.7%	83.1%
SII	0.715	0.676–0.753	1297.39	80.0%	59.5%	8.9%	98.4%	60.5%
AISI/Hb	0.714	0.675–0.752	70.19	76.0%	64.0%	9.5%	98.2%	64.6%
AISI	0.709	0.670–0.747	956.31	64.0%	74.5%	11.0%	97.7%	74.0%
GBS	0.673	0.633–0.713	7.0	76.0%	49.8%	7.0%	97.7%	51.0%
PLR	0.597	0.555–0.639	306.58	44.0%	85.4%	12.9%	96.9%	83.4%
Rockall	0.594	0.549–0.638	8.0	23.1%	97.4%	20.0%	97.8%	95.4%

Optimal cutoff values were determined using Youden index. AUC, area under the curve; CI, confidence interval; PPV, positive predictive value; NPV, negative predictive value; NLR, neutrophil-to-lymphocyte ratio; PLR, platelet-to-lymphocyte ratio; SII, systemic immune-inflammation index; SIRI, systemic inflammation response index; AISI, aggregate index of systemic inflammation; Hb, hemoglobin; GBS, Glasgow-Blatchford Score; ABC, Age–Blood–Comorbidity Score.

**Table 4:** Multivariable Logistic Regression Analysis for Mortality Prediction.

(A) Model 1: SIRI-Based Model			
Variable	Odds Ratio	95% CI	*p*-Value
Age	0.983	0.953–1.015	0.301
Sex (male)	0.421	0.153–1.159	0.094
Initial hemoglobin	1.2	0.991–1.453	0.062
Systolic BP	0.971	0.949–0.993	**0.011**
BUN	1.081	0.992–1.179	0.076
Albumin	0.06	0.018–0.203	**<0.001**
SIRI	1.102	1.022–1.188	**0.011**
**(B) Model 2: AISI-based Model**			
**Variable**	**Odds Ratio**	**95% CI**	***p*** **-Value**
Age	0.976	0.946–1.007	0.125
Sex (male)	0.361	0.129–1.007	0.052
Initial hemoglobin	1.212	1.005–1.461	**0.044**
Systolic BP	0.97	0.949–0.992	**0.009**
BUN	1.094	1.005–1.191	**0.038**
Albumin	0.054	0.016–0.184	**<0.001**
AISI (per 100 units)	1.036	1.012–1.061	**0.003**

(A): Model Statistics: McFadden’s R^2^ = 0.316, Log-Likelihood = −68.94, Likelihood ratio test *p* < 0.001. (B): Model Statistics: McFadden’s R^2^ = 0.321, Log-Likelihood = −68.42, Likelihood ratio test *p* < 0.001. BP, blood pressure; BUN, blood urea nitrogen; SIRI, systemic inflammation response index; AISI, aggregate index of systemic inflammation; CI, confidence interval. AISI odds ratio is calculated per 100-unit increase. Bold p-values indicate statistical significance (*p* < 0.05).

**Table 5:** Kaplan–Meier Survival Analysis Summary.

Score	Cutoff Value	Low Group (*n*)	Low Group Mortality (%)	High Group (*n*)	High Group Mortality (%)	Log-Rank *p*-Value	Test Statistic
SIRI	3.49	387	1.6%	143	13.3%	0.0	16.98
AISI	956.31	386	2.6%	144	10.4%	0.0553	3.67

Cutoff values were determined by ROC analysis using Youden index. SIRI, systemic inflammation response index; AISI, aggregate index of systemic inflammation. Log-rank test was used to compare survival curves between groups.

## Data Availability

The data presented in this study are available on request from the corresponding author due to privacy and ethical restrictions. The data are not publicly available as they contain patient information that could compromise patient confidentiality.
